# Neonatal Sepsis Episodes and Retinopathy of Prematurity in Very Preterm Infants

**DOI:** 10.1001/jamanetworkopen.2024.23933

**Published:** 2024-07-25

**Authors:** Kirsten Glaser, Christoph Härtel, Claus Klingenberg, Egbert Herting, Mats I. Fortmann, Christian P. Speer, Hans J. Stensvold, Zuzana Huncikova, Arild E. Rønnestad, Martin M. Nentwich, Andreas Stahl, Olaf Dammann, Wolfgang Göpel

**Affiliations:** 1 Division of Neonatology, Department of Women’s and Children’s Health, University of Leipzig Medical Center, Leipzig, Germany; 2 Department of Pediatrics, University Hospital of Würzburg, Würzburg, Germany; 3 Paediatric Research Group, Faculty of Health Sciences, University of Tromsø-Arctic University of Norway, Tromsø, Norway; 4 Department of Pediatrics and Adolescence Medicine, University Hospital of North Norway, Tromsø, Norway; 5 Department of Pediatrics, University Hospital of Schleswig-Holstein, Campus Lübeck, Lübeck, Germany; 6 Department of Neonatal Intensive Care, Clinic of Paediatric and Adolescent Medicine, Oslo University Hospital, Oslo, Norway; 7 Paediatric Department, Stavanger University Hospital, Stavanger, Norway; 8 Department of Clinical Science, University of Bergen, Bergen, Norway; 9 Department of Neonatal Intensive Care, Clinic of Paediatric and Adolescent Medicine, Oslo University Hospital, Oslo, Norway; 10 Medical Faculty, Institute for Clinical Medicine, University of Oslo, Oslo; 11 Department of Ophthalmology, University Hospital of Würzburg, Würzburg, Germany; 12 Department of Ophthalmology, University Medicine Greifswald, Greifswald, Germany; 13 Department of Public Health and Community Medicine, Tufts University School of Medicine, Boston, Massachusetts; 14 Department of Gynecology and Obstetrics, Hannover Medical School, Hannover, Germany; 15 Department of Neuromedicine and Movement Sciences, Norwegian University of Science and Technology, Trondheim, Norway

## Abstract

**Importance:**

Retinopathy of prematurity (ROP) is a major morbidity of preterm infants causing visual impairment, including blindness, for which timely treatment is vital and prevention is key. Increasing evidence suggests that exposure to neonatal sepsis contributes to ROP development.

**Objective:**

To investigate the association between neonatal sepsis and ROP in 2 large-scale cohorts of preterm infants born at less than 29 weeks’ gestation.

**Design, Setting, and Participants:**

This retrospective cohort study was conducted using data from the German Neonatal Network (GNN) and Norwegian Neonatal Network (NNN). The GNN involves 68 and the NNN includes 21 level III neonatal intensive care units. Participants were infants born at a gestation of 22 weeks and 0 days to 28 weeks and 6 days and enrolled in the GNN between January 1, 2009, and December 31, 2022, and NNN between January 1, 2009, and December 31, 2018. Data were analyzed from February through September 2023.

**Exposure:**

Single or multiple episodes of culture-proven sepsis.

**Main Outcomes and Measures:**

Any ROP and treatment-warranted ROP.

**Results:**

Among 12 794 infants in the GNN (6043 female [47.2%] and 6751 male [52.8%]; mean [SD] gestational age, 26.4 [1.5] weeks) and 1844 infants in the NNN (866 female [47.0%] and 978 male [53.0%]; mean [SD] gestational age, 25.6 [1.5] weeks), the mean (SD) birth weight was 848 (229) g and 807 (215) g, respectively. Any ROP was present in 6370 infants (49.8%) in GNN and 620 infants (33.6%) in NNN, and treatment-warranted ROP was present in 840 infants (6.6%) in GNN and 140 infants (7.6%) in NNN. In both cohorts, there were increasing rates of treatment-warranted ROP with each sepsis episode (no sepsis: 572 of 10 658 infants [5.4%] in GNN and 85 of 1492 infants (5.7%) in NNN; 1 episode: 190 of 1738 infants in GNN [10.9%] and 29 of 293 infants [9.9%] in NNN; 2 episodes: 53 of 314 infants in GNN [16.9%] and 13 of 49 infants [26.5%] in NNN; 3 episodes: 25 of 84 infants [29.8%] in GNN and 3 of 10 infants [30.0%] in NNN). After adjusting for multiple confounders in the GNN dataset, the number of sepsis episodes was associated with ROP and treatment-warranted ROP compared with 0 episodes (1 episode: adjusted odds ratio [aOR], 1.44 [95% CI, 1.27-1.63]; *P* < .001 and OR, 1.60 [95% CI, 1.31-1.96]; *P* < .001, respectively; 2 episodes: OR, 1.81 [95% CI, 1.35-2.42]; *P* < .001 and OR, 2.38 [95% CI, 1.68-3.37]; *P* < .001, respectively; 3 episodes: OR, 4.39 [95% CI, 2.19-8.78]; *P* < .001 and OR, 3.88 [95% CI, 2.29-6.55]; *P* < .001, respectively). These associations were confirmed for any ROP by propensity score matching (for example, the aOR with propensity score matching was 1.76 [95% CI, 1.54-2.02]; *P* < .001 for 1 episode vs 0 episodes and 1.58 [95% CI, 1.12-2.22]; *P* = .007 for 3 episodes vs 0 or 1 episode). In the NNN dataset, surgical NEC was associated with treatment-warranted ROP (multivariable analysis: aOR, 3.37 [95% CI, 1.78-6.37]; *P* < .001).

**Conclusions and Relevance:**

This study found that in the large-scale GNN cohort, recurrent culture-proven sepsis was associated with ROP and treatment-warranted ROP in infants born at less than 29 weeks.

## Key Points

**Question:**

Is neonatal sepsis associated with retinopathy of prematurity and treatment-warranted retinopathy in preterm infants born at less than 29 weeks of gestation?

**Findings:**

In this cohort study of 12 794 infants in the German Neonatal Network and 1844 infants in the Norwegian Neonatal Network, exposure to recurrent sepsis was associated with the development of retinopathy of prematurity and with the need for treatment. The number of sepsis episodes was associated with an increase in odds of retinopathy of prematurity.

**Meaning:**

These findings suggest that recurrent culture–proven sepsis ought to be acknowledged as a preventable risk factor associated with retinopathy of prematurity in very preterm infants.

## Teaser

This cohort study investigates the association of neonatal sepsis episodes with retinopathy of prematurity, including treatment-warranted cases, among very preterm infants in Germany and Norway.

## Introduction

Retinopathy of prematurity (ROP) is a major morbidity of preterm infants characterized by arrested retinal vascularization followed by excessive vasoproliferation.^1,2,3^ Timely diagnosis and treatment are essential to prevent irreversible visual impairment or blindness. Low gestational age (GA), low birth weight, poor postnatal growth, and exposure to hyperoxia and hypoxia are well-known risk factors.^1,3,4^ With improved survival of very preterm infants and despite an ongoing search for optimized strategies to control oxygen saturation and supply,^5,6,7^ rates of ROP have not declined.^2,3,8^ The incidence of treatment-warranted ROP in overall screening cohorts of very preterm infants in high-income countries ranges from 2% to 6%.^3,9^ In selected high-risk cohorts, an incidence of 4% to 15%, sometimes as high as 30%, is reported.^8,9^ Laser photocoagulation and intravitreal antivascular endothelial growth factor (VEGF) injections are the most prevalent treatment modalities.^1,3,10,11^ Of note, even infants with timely treatment or ROP stages below treatment thresholds are at increased risk of permanent visual impairment.^1,3^ Therefore, a better understanding of actionable risk factors for prevention and early diagnosis remains paramount.

Neonatal sepsis has emerged as a potential postnatal risk factor associated with ROP in recent years.^12,13,14,15,16,17,18^ Data from observational studies and animal models suggest a contributing role of infection and inflammation in the pathogenesis of ROP.^19,20,21,22,23^ Small prospective studies support these findings.^24,25^ Whether early-onset sepsis (EOS) and (recurrent) late-onset sepsis (LOS) are associated with different changes in ROP risk remains unclear.^15,26,27^ In this study, we used data from the German Neonatal Network (GNN) and Norwegian Neonatal Network (NNN) to test whether very preterm infants who survived 1 or more episode of culture-proven sepsis were at increased risk of developing ROP or treatment-warranted ROP.

## Methods

### Study Population

The GNN is a population-based observational multicenter study involving 68 level III neonatal intensive care units (NICUs) in Germany investigating risks and complications of infants with very low birth weight born at a gestation of 22 weeks and 0 days to 36 weeks and 6 days. This study includes infants born at 22 weeks and 0 days to 28 weeks and 6 days enrolled between January 1, 2009, and December 31, 2022. Written informed consent by parents or guardians was mandatory for enrollment. Predefined clinical data were recorded on case report forms, coded, and stored centrally at the coordinating center in Lübeck, Germany. All infants born at less than 29 weeks admitted to participating centers were eligible. Exclusion criteria were lethal malformation and lack of written consent. All study parts of the GNN were approved by the ethics committee of the University of Lübeck and local ethics committees of all participating centers. GNN review and satisfaction of informed consent extend to this study. Data quality was ensured by annual on-site monitoring. The NNN is a national population-based registry comprising all 21 NICUs across 4 health trust regions. Reporting to the registry is compulsory without consent within current national legislation and includes daily registrations of all investigations, treatments, and diagnoses of all infants admitted to 1 or several Norwegian NICUs until final discharge.^28,29^ The NNN cohort in this study includes infants born at a gestation of 22 weeks and 0 days to 28 weeks and 6 days between January 1, 2009, and December 31, 2018. All study parts of the NNN were approved by the regional committee for medical and health research ethics. NNN review and exemption for informed consent extend to this study. Our study followed the Strengthening the Reporting of Observational Studies in Epidemiology (STROBE) reporting guideline for cohort studies.

### Ophthalmological Screening and ROP Definition

In Germany and Norway, standardized ROP screening during the study period included all preterm infants born at less than 32 weeks’ gestation (<31 weeks in Germany from 2020), with birth weight less than 1500 g independent of the need of supplemental oxygen, or both characteristics. Moreover, screening is recommended in Germany for infants born at a gestation of 31 weeks and 0 days to 36 weeks and 6 days with a requirement of supplemental oxygen for more than 5 days or the presence of severe comorbidities, such as necrotizing enterocolitis (NEC), bronchopulmonary dysplasia, postnatal sepsis, or severe anemia requiring red blood cell (RBC) transfusion.^30^ In GNN and NNN centers, screening started at ages 5 to 6 weeks and not before age 31 weeks. Examinations were performed weekly or biweekly depending on individual findings and were continued until physiologic vascularization reached zone III, preexisting ROP showed continuous regression, or both conditions were met. ROP was defined according to the International Classification of Retinopathy of Prematurity consensus statement.^31^ Severity was designated by zone, stage, and circumferential extent assessed by sectors and the absence or presence of plus disease.^31^ Treatment-warranted ROP referred to aggressive ROP, any stage ROP in zone I with plus disease, ROP 3 in zone I, or ROP 3+ in zone II according to national guidelines that required laser therapy, cryotherapy, or anti-VEGF treatment.^30,32^

### Sepsis Definition

Neonatal sepsis was defined as blood culture–proven sepsis identified by clinical sepsis criteria and detection of a causative pathogen in 1 or more blood cultures according to criteria of the German National Nosocomial Infection Surveillance System in Preterm Infants (NEO-KISS).^33^ EOS was defined as sepsis occurring within the first 72 hours of life, while late-onset sepsis (LOS) was defined as sepsis episode after 72 hours of life.^34,35^ The analysis of 1, 2, or 3 episodes included EOS or LOS episodes. Neither the GNN nor the NNN dataset provided data on the respective causative agent.

### Definitions of Clinical Parameters

GA was calculated from the best obstetric estimate according to early prenatal ultrasonography and obstetric examination. Small for GA was defined as a birth weight less than the 10th percentile according to GA; inborn was defined as birth in a network center. Clinical chorioamnionitis, with data available only from GNN, was documented by the attending obstetrician as cause of preterm birth based on maternal fever plus fetal tachycardia, a white blood cell count greater than 15 000/μL, or a foul-smelling discharge. Days on supplemental oxygen and days on mechanical ventilation or continuous positive airway pressure (CPAP) referred to the number of complete days of exposure. Bronchopulmonary dysplasia was defined as requirement of oxygen therapy for at least 28 days and classified as mild, moderate, or severe according to the need for supplemental oxygen at 36 weeks’ postmenstrual age.^36^ Intraventricular hemorrhage was diagnosed according to Papile classification.^37^ Cystic periventricular leukomalacia was defined as periventricular, cystic white matter lesions. Both entities were diagnosed using cranial ultrasonography. NEC requiring surgery corresponded to clinical NEC classified as Bell stage II or III with the need for laparotomy^38^ and macroscopic diagnosis of NEC.

### Statistical Analysis

Study groups were compared using univariable analyses, including the Mann-Whitney *U* test for continuous and 2-sided χ^2^ test for categorical variables. The primary analysis used stepwise multivariable logistic regression to adjust for confounders, accounting for the multihit sequence of ROP development and the hypothesized sequential nature of inflammation-induced perinatal and postnatal retinal injury.^2,39^ Variables offered to model 1 referred to prenatal and perinatal characteristics, while covariates offered to model 2 were those significant at *P* < .10 in model 1 plus surrogate markers of a poor clinical course. Logistic regression model 3 added RBC transfusions and platelet and fresh frozen plasma transfusions to analyses (eTables 1 and 2 in Supplement 1). Correlation analysis was used to confirm variable correlation with ROP and treatment-warranted ROP. Secondary analysis used conditional regression analysis performed in propensity score–matched infants. Odds ratios (ORs) and adjusted ORs (aORs) with 95% CIs were reported. A 2-tailed *P* value < .05 was considered statistically significant. Missing data were not imputed. Analyses were performed using SPSS statistical software version 28.0 (IBM) and R statistical software version 4.3.3 (R Project for Statistical Computing). Data were analyzed from February until September 2023.

## Results

A total of 12 794 preterm infants from the GNN born at less than 29 weeks’ gestation and screened for ROP were included (6043 female [47.2%] and 6751 male [52.8%]; mean [SD] GA, 26.4 [1.5] weeks) (Table 1; eFigure 1 in Supplement 1), while 1844 of 2244 admitted infants from the NNN (82.2%) born at less than 29 weeks’ gestation had their eyes examined for ROP and thus constituted the Norwegian cohort (866 female [47.0%] and 978 male [53.0%]; mean [SD] GA, 25.6 [1.5] weeks) (Table 2; eFigure 2 in Supplement 1). The mean (SD) birth weight was 848 (229) g in the GNN and 807 (215) g in the NNN.

**Table 1.** Clinical Characteristics of Infants in the German Neonatal Network

Characteristic	Infants, No. (%) (N = 12 794)		
	No ROP (n = 6424)	Any ROP (n = 6370)	*P* value
Gestational age, mean (SD), wk	27.03 (1.35)	25.84 (1.61)	<.001
Birth weight, mean (SD), g	931 (232)	765 (225)	<.001
Sex			
Female	2988 (46.5)	3056 (48.0)	.10
Male	3436 (53.5)	3317 (52.0)	
Multiple birth	2031 (31.6)	2007 (31.5)	.96
SGA status	519 (8.1)	1142 (17.9)	<.001
Inborn status	6053 (97.1)	5973 (93.8)	.33
Antenatal steroid use	5960 (92.8)	5633 (88.4)	<.001
Clinical chorioamnionitis	1794 (27.9)	1795 (28.2)	.16
Apgar score at 5 min, mean (SD)	7 (1)	7 (2)	.01
LISA	2968 (46.2)	2660 (41.8)	<.001
Days on mechanical ventilation or CPAP, mean (SD)	43 (28)	69 (40)	<.001
Days on supplemental oxygen, mean (SD)	33 (34)	63 (50)	<.001
Maximum FiO_2_ in the first 12 h of life, mean (SD)	43 (22)	48 (25)	<.001
FiO_2_ 0.21-029 in the first 12 h of life, mean (SD)	1832 (28.5)	1412 (22.1)	
FiO_2_ 0.3-1.0 in the first 12 h of life, mean (SD)	4518 (70.3)	4655 (73.1)	
Inotropes given in the first 24 h	636 (10.0)	1017 (16.0)	<.001
Days until full enteral feeds, mean (SD)	15.92 (11.33)	22.05 (18.86)	<.001
Neonatal sepsis	752 (11.7)	1387 (21.8)	<.001
Sepsis episodes			
1	656 (10.2)	1082 (17.0)	<.001
2	82 (1.3)	232 (3.6)	
3	11 (0.2)	73 (1.1)	
Early-onset sepsis	83 (1.3)	87 (1.4)	.58
Late-onset sepsis	669 (10.4)	1300 (20.4)	<.001
Any IVH	1265 (19.7)	2012 (31.7)	<.001
IVH grade			
I	553 (8.6)	644 (10.1)	<.001
II	351 (5.5)	560 (8.8)	
III	188 (2.9)	415 (6.5)	
IV	173 (2.7)	387 (6.1)	
cPVL	183 (2.9)	361 (5.7)	<.001
NEC requiring surgery	113 (1.8)	358 (5.6)	<.001

Abbreviations: CPAP, continuous positive airway pressure; cPVL, cystic periventricular leukomalacia; FiO_2_, fraction of inspired oxygen; IVH, intraventricular hemorrhage; LISA, less invasive surfactant administration; NEC, necrotizing enterocolitis; ROP, retinopathy of prematurity; SGA, small for gestational age.

**Table 2.** Characteristics of Infants in the Norwegian Neonatal Network

Characteristic	Infants, No. (%) (N = 1844)		*P* value
	Treatment-warranted ROP (n = 140)	ROP stages < treatment thresholds (n = 1704)	
Gestational age, mean (SD), wk	24.95 (1.57)	26.46 (1.44)	<.001
Birth weight, mean (SD), g	695 (195)	919 (235)	<.001
Sex			
Female	57 (40.7)	809 (47.5)	.13
Male	83 (59.3)	895 (52.5)	
SGA status	42 (30.0)	384 (22.5)	.05
Antenatal steroid use	134 (95.7)	1514 (88.8)	.007
Apgar score at 5 min, median (IQR)	7 (5-8)	7 (6-9)	<.001
Days on mechanical ventilation or CPAP, median (IQR)	83 (67-103)	51 (38-70)	<.001
Days on supplemental oxygen, median (IQR)	97 (77-131)	59 (43-83)	<.001
Days until full enteral feeds, median (IQR)	13 (10-23)	10 (7-13)	<.001
Neonatal sepsis	45 (32.1)	307 (18.0)	<.001
Sepsis episodes			
1	29 (20.7)	264 (15.5)	.12
2	13 (9.3)	36 (2.1)	<.001
3	3 (2.1)	7 (0.4)	.04
IVH grade 3-4	12 (8.6)	92 (5.4)	.13
cPVL	7 (5.0)	77 (4.5)	.83
NEC requiring surgery	20 (14.3)	38 (2.2)	<.001

Abbreviations: CPAP, continuous positive airway pressure; cPVL, cystic periventricular leukomalacia; IVH, intraventricular hemorrhage; NEC, necrotizing enterocolitis; ROP, retinopathy of prematurity; SGA, small for gestational age.

There were 2039 GNN infants not included in analyses due to missing data on ophthalmological screening (eFigure 1 in Supplement 1). The prevalence of any ROP in the GNN group was 6370 infants (49.8%), comprising 2697 infants with stage 1 disease (21.1%), 2171 infants with stage 2 disease (17.0%), 1282 infants with stage 3 disease (10.0%), and 31 infants with stage 4 disease or higher (0.2%). There were 189 infants (1.5%) diagnosed with ROP but for whom classification was not available (eFigure 1 in Supplement 1). ROP was graded as treatment-warranted ROP in 840 infants (6.6%). In the overall German study cohort, we documented a history of 1 sepsis episode in 1738 infants (13.6%), 2 episodes in 314 infants (2.5%), and 3 episodes in 84 infants (0.7%). The Figure displays the percentages of infants with ROP and treatment-warranted ROP stratified by GA and the number of sepsis episodes.

**Figure.**
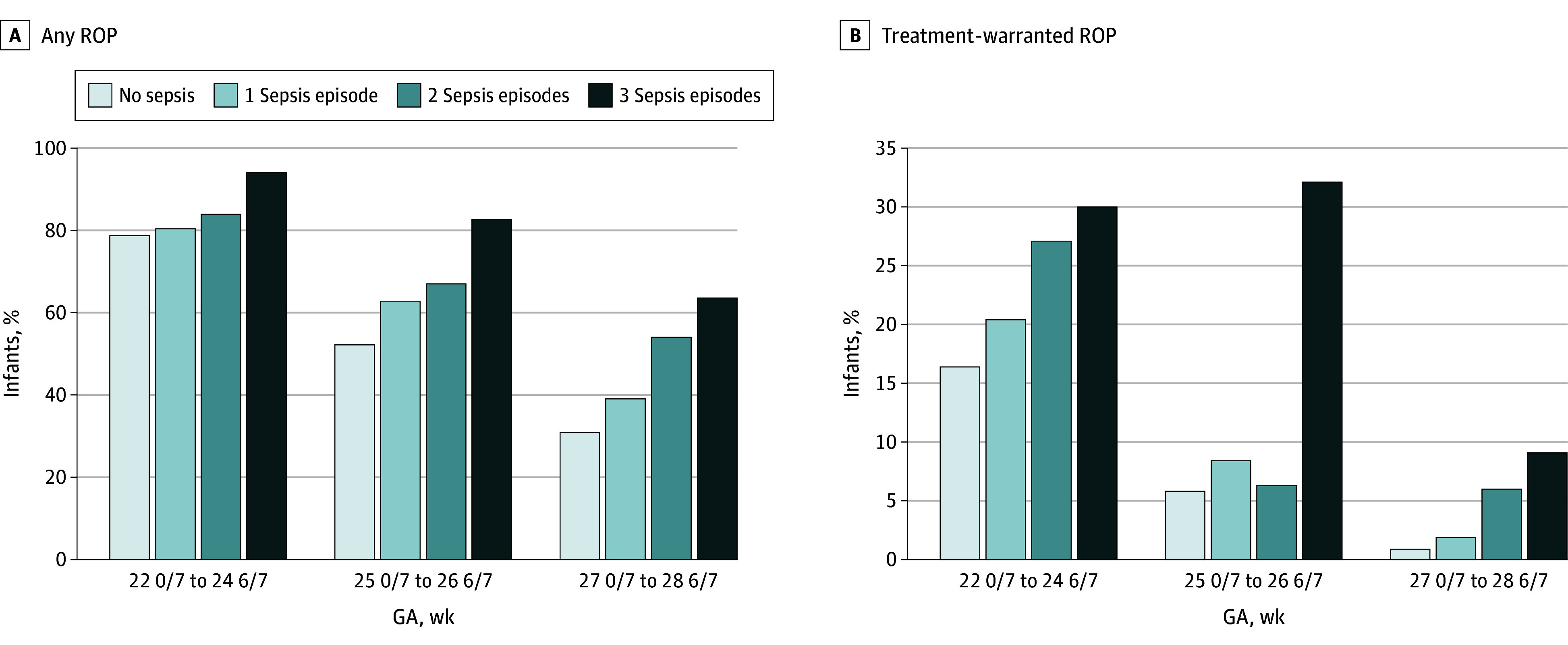
Rates of Retinopathy of Prematurity (ROP) in the German Neonatal Network The total number of infants in the respective gestational age (GA) strata and sorted by number of sepsis episodes was as follows: For infants with a GA of 22 weeks and 0 days to 24 weeks and 6 days, there were 1831 infants with 0, 588 infants with 1, 155 infants with 2, and 50 infants with 3 episodes of sepsis. For infants with a GA of 25 weeks and 0 days to 26 weeks and 6 days, there were 3776 infants with 0, 702 infants with 1, 112 infants with 2, and 28 infants with 3 episodes of sepsis. For infants with a GA of 27 weeks and 0 days to 28 weeks and 6 days, there were 5023 infants with 0, 468 infants with 1, 50 infants with 2, and 11 infants with 3 episodes of sepsis.

In univariable analysis in the GNN, the prevalence of neonatal sepsis (752 infants [11.7%] vs 1387 infants [21.8%]; *P* < .001) and the number of sepsis episodes (eg, 656 infants [10.2%] vs 1082 infants [17.0%] with 1 episode; *P* < .001) differed between infants with and without ROP (Table 1). EOS was not associated with ROP risk. On the contrary, 1 sepsis episode occurring as LOS and 2 to 3 episodes of sepsis as EOS plus LOS or recurrent LOS were associated with increased ROP risk (Table 1). Comparisons further suggested that infants with ROP had a lower GA and birth weight and were more likely to be small for GA. Moreover, this group spent more days on mechanical ventilation or CPAP and supplemental oxygen, had a higher maximum fraction of inspired oxygen in the first 12 hours, needed more days until full enteral feeds, and was more likely to have a history of inotropes in the first 24 hours and of intraventricular hemorrhage, periventricular leukomalacia, and NEC requiring surgery. Antenatal steroids and less invasive surfactant administration were associated with reduced ROP risk (Table 1).

Using a stepwise logistic regression approach, we adjusted for known and probable risk factors associated with ROP development. The number of sepsis episodes as a covariate was associated with increased odds of ROP in GNN vs 0 episodes (1 episode: aOR, 1.44 [95% CI, 1.27-1.63]; *P* < .001; 2 episodes: aOR, 1.81 [95% CI, 1.35-2.42]; *P* < .001; 3 episodes: aOR, 4.39 [95% CI, 2.19-8.78]; *P* < .001) (Table 3; eFigure 3 in Supplement 1). A history of NEC requiring surgery was also associated with increased odds of ROP (eFigure 3 in Supplement 1).

**Table 3.** Association of Neonatal Sepsis With ROP in the German Neonatal Network

Sepsis episodes, No.	Stepwise multivariable logistic regression modeling^a^				Conditional regression analysis in propensity-score matched infants^b^			
	Any ROP		Treatment-warranted ROP		Any ROP		Treatment-warranted ROP	
	aOR (95% CI)^c^	*P* value	aOR (95% CI)^c^	*P* value	aOR (95% CI)^c^	*P* value	aOR (95% CI)^c^	*P* value
1								
Model 1	1.44 (1.27-1.63)	<.001	1.60 (1.31-1.96)	<001	1.76 (1.54-2.02)	<.001	1.90 (1.52-2.37)	<.001
Model 2	1.23 (1.08-1.40)	.002	1.26 (1.02-1.55)	.03				
Model 3	1.20 (1.02-1.30)	.05	1.14 (0.92-1.40)	.24				
2								
Model 1	1.81 (1.35-2.42)	<.001	2.38 (1.68-3.37)	<.001	1.58 (1.12-2.22)	.007	1.41 (0.95-2.11)	.09
Model 2	1.36 (1.01-1.84)	.05	1.66 (1.16-2.38)	.006				
Model 3	1.27 (0.94-1.71)	.12	1.58 (1.11-2.25)	.01				
3								
Model 1	4.39 (2.19-8.78)	<.001	3.88 (2.29-6.55)	<.001	1.58 (1.12-2.22)	.007	1.41 (0.95-2.11)2	.09
Model 2	2.06 (1.04-4.10)	.04	1.84 (1.04-3.27)	.04				
Model 3	1.89 (0.96-3.75)	.07	1.63 (0.93-2.87)	.09				

Abbreviations: aOR, adjusted odds ratio; ROP, retinopathy of prematurity.

^a^ Stepwise multivariable regression modeling included 6424 infants without ROP and 6370 infants diagnosed with any ROP. See the eMethods in Supplement 1 for descriptions of each model.

^b^ Conditional regression analysis in propensity score-matched infants included: 4244 infants (2122 matched pairs of study infants without sepsis and infants with 1 sepsis episode) for the analysis of 1 sepsis episode and 800 infants (400 matched pairs of infants without sepsis or 1 sepsis episode and infants with 2-3 episodes) for the analyses of 2 or 3 sepsis episodes. Both pairs showed full matching defined as no differences between demographic and clinical characteristics. Propensity score matching was performed using gestational age, small for gestational age, sex, clinical chorioamnionitis, and presence of central venous line as variables, all associated with the individual’s risk of sepsis. Subsequent conditional regression analysis adjusted for variables included in the stepwise logistic regression modeling.

^c^ The reference group for all aORs is infants with 0 sepsis episodes except for analyses of 2 or 3 sepsis episodes in propensity score–matched infants, for which the reference group was infants with 0 or 1 sepsis episode.

Given the outcomes associated with treatment-warranted ROP, we compared clinical characteristics among infants with this diagnosis and infants with no ROP or less severe ROP stages. These groups differed significantly in the number of sepsis episodes and parameters suggestive of a more complicated clinical course. Using the stepwise regression modeling approach previously described, we hypothesized an association between the number of sepsis episodes included as a categorical covariate and treatment-warranted ROP. Models 1 and 2 documented an association of 1 episode (eg, model 1: aOR, 1.60 [95% CI, 1.31-1.96]; *P* < .001), 2 episodes (eg, model 1: aOR, 2.38 [95% CI, 1.68-3.37]; *P* < .001), and 3 episodes (eg, model 1: aOR, 3.88 [95% CI, 2.29-6.55]; *P* < .001) of sepsis with odds of treatment-warranted ROP vs 0 episodes (Table 3; eFigure 3 in Supplement 1). In model 3, we found an association of 2 sepsis episodes with treatment-warranted ROP (aOR vs 0 episodes, 1.58 [95% CI, 1.11-2.25]; *P* = .01) (Table 3; eFigure 3 in Supplement 1). Higher GA, female sex, and clinical chorioamnionitis were associated with decreased odds of treatment-warranted ROP. In contrast, NEC requiring surgery and RBC and platelet transfusions were associated with increased odds of treatment-warranted ROP (Table 3; eFigure 3 in Supplement 1).

Given the interrelated nature of variables included in this modeling, we performed propensity score matching as an alternative statistical approach. In 2122 matched pairs of infants without sepsis and infants with 1 sepsis episode, sepsis was associated with ROP (aOR, 1.76 [95% CI, 1.54-2.02]; *P* < .001) and treatment-warranted ROP (aOR, 1.90 [95% CI, 1.52-2.37); *P* < .001) compared with 0 episodes. In the smaller group of 400 matched pairs of infants without sepsis or with 1 sepsis episode and patients with 2 (aOR, 1.58 [95% CI, 1.12-2.22]; *P* = .007) and 3 (aOR, 1.58 [95% CI, 1.12-2.22]; *P* = .007) sepsis episodes, recurrent sepsis was associated with increased odds of ROP vs 0 or 1 episode (Table 3). There were increasing rates of treatment-warranted ROP with each sepsis episode (no sepsis: 572 of 10 658 infants [5.4%]; 1 episode: 190 of 1738 infants [10.9%]; 2 episodes: 53 of 314 infants [16.9%]; 3 episodes; 25 of 84 infants [29.8%])

Among infants in the NNN, any ROP was present in 620 infants (33.6%). ROP was graded as treatment-warranted ROP in 140 infants (7.6%). In the overall Norwegian study cohort, we documented a history of 1 sepsis episode in 293 infants (15.9%), 2 episodes in 49 infants (2.7%), and 3 episodes in 10 infants (0.5%). Characteristics of infants with treatment-warranted ROP compared with those not treated are presented in Table 2. In univariable analysis, similar findings as in the GNN cohort were detected (Table 4), with an increase in the risk of treatment-warranted ROP with more episodes of sepsis (no sepsis: 85 of 1492 infants [5.7%]; 1 episode: 29 of 293 infants [9.9%]; 2 episodes 13 of 49 infants [26.5%]; 3 episodes: 3 of 10 infants [30.0%]). In a multivariable model adjusting for other variables associated with large increases in risk of treatment-warranted ROP, we no longer observed an association between episodes of sepsis and risk for treatment-warranted ROP in the NNN cohort. In all analyses, NEC requiring surgery remained associated with the greatest increase in risk of treatment-warranted ROP, with aORs ranging from 3.37 (95% CI, 1.78-6.37; *P* < .001) in multivariable analysis to 7.30 (95% CI, 4.12-12.95; *P* < .001) in univariable analysis compared with infants without a history of NEC requiring surgery (Table 4).

**Table 4.** Association of Neonatal Morbidities With Treatment-Warranted ROP in the Norwegian Neonatal Network

Variable^a^	Univariable analysis, OR (95% CI)	*P* value	Multivariable analysis, aOR (95% CI)	*P* value
Neonatal sepsis	2.16 (1.48-3.14)	<.001	0.97 (0.64-1.47)	.88
Sepsis episodes, No.^b^				
1	1.61 (1.04-2.50)	.03	0.77 (0.47-1.23)	.29
2	5.30 (2.72-10.34)	<.001	1.96 (0.94-4.10)	.07
3	6.29 (1.60-24.73)	.008	1.89 (0.40-8.83)	.42
NEC requiring surgery	7.30 (4.12-12.95)	<.001	3.37 (1.78-6.37)	<.001
SGA	1.47 (1.01-2.15)	.05	1.53 (1.01-2.32)	.04
GA, wk^c^	0.54 (0.48-0.60)	<.001	0.63 (0.54-0.72)	<.001
Severe BPD	3.84 (2.69-5.46)	<.001	1.80 (1.21-2.67)	.004
Apgar score at 5 min <7	0.58 (0.41-0.82)	.002	1.02 (0.71-1.52)	.83

Abbreviations: aOR, adjusted odds ratio; BPD, bronchopulmonary dysplasia; GA, gestational age; NEC, necrotizing enterocolitis; OR, odds ratio; ROP, retinopathy of prematurity; SGA, small for gestational age.

^a^ Variables not associated with treatment-warranted ROP in univariable analysis were female sex, intraventricular hemorrhage grade 3 to 4, and cystic periventricular leukomalacia.

^b^ The reference group was infants with 0 sepsis episodes.

^c^ Outcomes were per 1-week increase in GA.

## Discussion

In this cohort study, we found an association of neonatal sepsis and recurrent episodes with an increased risk of ROP and treatment-warranted ROP in a large dataset from Germany. These findings are in keeping with data from previous retrospective and a few prospective studies, all with smaller sample sizes, reporting an association of 1 and more infectious episodes with increased risk of ROP and higher-stage ROP requiring therapy.^15,24,25,26,27,40^ To the best of our knowledge, our study is the first to describe a dose-response association between the number of sepsis episodes and increases in the odds of ROP and treatment-warranted ROP. Notably, in the GNN dataset culture-proven sepsis was associated with a greater increase in risk of ROP and treatment-warranted disease than were GA, birth weight, and days on supplemental oxygen, although these have been widely acknowledged as major risk factors.^1,3,4,8,25^ In the smaller cohort from Norway, there was no association between neonatal sepsis and treatment-warranted ROP after adjusting for confounders. Instead the inflammatory condition of NEC was associated with the greatest increase in risk of treatment-warranted ROP in the NNN cohort.

This observational study found associations and results cannot be used to conclude causation.^41^ However, observations of higher rates of ROP in preterm infants with sepsis, including fungal sepsis, date back more than 25 years,^40,42,43^ but it is only in the past decade that neonatal sepsis has been discussed as an independent risk factor associated with ROP rather than a covariate reflecting a poor overall clinical course.^12,13,14,15,16,17,39^ Underlying mechanisms remain to be elucidated.^39,44^ Neonatal sepsis may modify the second phase of ROP characterized by hypoxia and compensatory growth factor–induced aberrant retinal vascularization^45^ or may contribute to the arrest of vascularization in phase 1 and pathological neovascularization in phase 2.^46^ Observational findings of higher incidences of ROP in infants with increased markers of inflammation, along with animal models suggest that inflammatory mediators are negatively associated with retinal vessel development in a direct or indirect way and may be associated with permanently compromised retinal function.^20,21,22,23,47,48,49,50,51^ Inflammation-induced reactive oxygen species and oxidative stress, as well as inflammation-induced VEGF expression, microglia activation, and retinal ganglion cell death have been discussed as causative mechanisms.^39,44,47,48^ In rat models, microglia-derived interleukin (IL)-1β was found to induce retinal ganglion cell death and breakdown of the blood-retina barrier,^47,52^ while application of IL-1 receptor antagonists attenuated vasoobliteration.^53^ In interaction with growth factors, a number of other leukocyte and microglia-derived cytokines and chemokines have been implicated in the disturbance of retinal vasculature.^54,55^ Ongoing basic research may help to identify therapeutics modulating inflammation-driven retinal damage.^54,55^ Notably, animal models showed that intravitreal administration of noncoding RNAs and gut microbiota modulation were potentially effective in regulating the course of ROP.^55^ The latter approach is in line with increasing evidence for a gut-retina axis and increasing interest in understanding the potential associations of infections, use of antibiotics, and gut dysbiosis with the development of retinopathy.^56^ Ultimately, hemodynamic and respiratory instability, frequently accompanying sepsis and NEC, may impair tissue perfusion and oxygen saturation and cause retinal ischemia at a vulnerable stage of development.^39,57^ In summary, neonatal sepsis and systemic inflammation observed in NEC may be closely intertwined in a multihit sequence of ROP.^19,39,44^ In this context, it may be worthwhile to discriminate whether multiple sepsis episodes are surrogates for true new infections or instead for recurrent episodes of the same origin or correlative of sustained inflammation with an undulating course.

While some studies have indicated that LOS but not EOS was associated with increased risk of ROP,^15,26,58^ others found an association for EOS and yet others for infections early and later in life.^27,59^ In a meta-analysis,^16^ EOS and LOS were associated with severe ROP. However, only 3 of 34 studies discriminated between EOS and LOS.^16^ In our study, EOS alone was not associated with ROP or treatment-warranted ROP, while repetitive LOS episodes or EOS followed by LOS were associated. Whether prenatal exposure to inflammation, namely chorioamnionitis, and subsequent fetal inflammation are associated with increased risk of later ROP has not been conclusively answered.^4,46,49,60,61^ An inflammation-induced prenatal sensitization of the immature retina to secondary injurious hits has been discussed.^1^ Two meta-analyses^22,62^ evaluating chorioamnionitis and ROP development found no association with ROP or treatment-warranted ROP when adjusting for confounders. A 2024 meta-analysis^63^ described an association between histological chorioamnionitis and the development of severe ROP. Notably, these findings were confounded by GA.^63^ On the contrary, a 2023 study^64^ in 182 placental tissues of infants born at less than 32 weeks and with a birth weight less than 1500 g that included comprehensive histopathological examinations and plasma protein analyses found that acute placental inflammation was associated with reduced risk of ROP. In addition, a 2024 nation-based cohort study^65^ from Japan comprising 38 013 preterm infants born at 1500 g or less or less than 32 weeks of gestation found a reduced risk of severe ROP in infants with severe histological chorioamnionitis. In keeping with these findings, a history of clinical chorioamnionitis was associated with decreased odds of ROP and treatment-warranted ROP in our study. Data on histological exams of placentas were not available.

The prevalence of any ROP in GNN and NNN study cohorts was 49.8% and 33.6%, respectively. This is in line with a notably wide range of 27% to 91% of any ROP reported in other multicenter and nationwide studies in select groups of preterm infants at high risk.^8,25,66,67,68,69^ Whether diverging ranges reflect differences in active care and survival of the most premature infants, mirror varying screening routines, or indicate risk factors not sufficiently addressed in some centers remains to be elucidated. Regardless of the case and against the background of an ever-decreasing GA of today’s NICU population, these numbers highlight the need to continue deciphering underlying mechanisms of ROP and disease progression and to improve prevention. Predictive algorithms of ROP have been developed aiming at individualizing risk prediction and early identification of infants at the highest risk.^70,71,72^ So far, neonatal sepsis has not been included in any of these scores.

### Limitations

This study has some limitations. These are partly related to the observational design that does not allow for any causal conclusion and may result in residual confounding. In addition to unknown differences in baseline characteristics, differences in clinical care or poor overall clinical course may affect rates of ROP and treatment-warranted ROP. Addressing these concerns, we controlled for several known and probable prenatal, perinatal, and infant risk factors, including NEC requiring surgery and RBC transfusions, which were also associated with ROP and treatment-warranted ROP. The latter findings are in keeping with previous investigations of our group and other cohort and network studies describing RBC transfusions and the concomitant fetal hemoglobin reduction as an independent risk factor associated with ROP and progression to higher-stage disease.^73,74,75,76^ On the contrary, our analyses confirmed previous findings of protective associations of antenatal steroids with ROP development.^77^ Moreover, female sex was associated with decreased odds of treatment-warranted ROP. This observation may be in line with the frequently observed phenomenon that more male than female infants are treated for ROP.^8,78^ It is imperative to mention that there may be potential confounders that were missed in applied regression models. The GNN dataset registered largely stable rates of ROP between 2009 and 2022. However, the evolution of neonatal care of very preterm infants in this 14-year period could have biased the results. Additionally, our study was limited by the relatively small sample size of infants with treatment-warranted ROP despite the use of 2 large-scale datasets spanning a 14-year and 10-year observation period. Findings in both datasets were in the same direction. However, large and well-powered prospective studies are needed to confirm our findings.

## Conclusions

In this large-scale cohort study, culture-proven neonatal sepsis and recurrent sepsis episodes in particular were associated with ROP and treatment-warranted ROP in preterm infants born at less than 29 weeks. To the best of our knowledge, this is the first investigation to reveal a direct association between the number of sepsis episodes and OR increases for ROP and treatment-warranted ROP. Further investigations need to explore whether this association is causal and define underlying mechanisms of potentially inflammation-driven retinal morbidity. However, these findings highlight once more the importance of sepsis prevention in preterm infants. Additionally, our results emphasize the necessity to consider neonatal sepsis as a risk factor in ROP screening policies in very preterm infants.

## Notes

Supplement 1. **eFigure 1.** Study Flowchart of Infants Enrolled in the German Neonatal Network **eFigure 2.** Study Flowchart of Infants Enrolled in the Norwegian Neonatal Network **eFigure 3.** Variables Independently Associated With ROP in GNN in Stepwise Multivariable Regression Analyses **eTable 1.** Multivariable Logistic Regression Modeling Testing for Association Between Neonatal Sepsis and Development of Any ROP in Very Preterm Infants in the GNN **eTable 2.** Multivariable Logistic Regression Modeling Testing for Association Between Neonatal Sepsis and Development of Treatment-Warranted ROP in the GNN **eMethods.** Description of Models Used in Analysis of Association With ROP and Treatment-Warranted ROP in GNN

Supplement 2. **Nonauthor Collaborators.** German Neonatal Network and Norwegian Neonatal Network Investigators

Supplement 3.
Data Sharing Statement
