# Supplementary material for: Neonatal Sepsis Episodes and Retinopathy of Prematurity in Very Preterm Infants
Source: JAMA Netw Open. 2024 Jul 25;7(7):e2423933. doi: 10.1001/jamanetworkopen.2024.23933 (PMC11273231; doi:10.1001/jamanetworkopen.2024.23933)
Supplement: Supplement 1. — eFigure 1. Study Flowchart of Infants Enrolled in the German Neonatal Network eFigure 2. Study Flowchart of Infants Enrolled in the Norwegian Neonatal Network eFigure 3. Variables Independently Associated With ROP in GNN in Stepwise Multivariable Regression Analyses eTable 1. Multivariable Logistic Regression Modeling Testing for Association Between Neonatal Sepsis and Development of Any ROP in Very Preterm Infants in the GNN eTable 2. Multivariable Logistic Regression Modeling Testing for Association Between Neonatal Sepsis and Development of Treatment-Warranted ROP in the GNN eMethods. Description of Models Used in Analysis of Association With ROP and Treatment-Warranted ROP in GNN [file jamanetwopen-e2423933-s001.pdf]

Supplemental Online Content

Glaser K, Härtel C, Klingenberg C, et al; for the German Neonatal Network and the Norwegian Neonatal Network Investigators. Neonatal sepsis episodes and retinopathy of prematurity in very preterm infants. *JAMA Netw Open*. 2024;7(7):e2423933. doi:10.1001/jamanetworkopen.2024.23933

eFigure 1. Study Flowchart of Infants Enrolled in the German Neonatal Network

eFigure 2. Study Flowchart of Infants Enrolled in the Norwegian Neonatal Network

eFigure 3. Variables Independently Associated With ROP in GNN in Stepwise Multivariable Regression Analyses

eTable 1. Multivariable Logistic Regression Modeling Testing for Association Between Neonatal Sepsis and Development of Any ROP in Very Preterm Infants in the GNN

eTable 2. Multivariable Logistic Regression Modeling Testing for Association Between Neonatal Sepsis and Development of Treatment-Warranted ROP in the GNN

eMethods. Description of Models Used in Analysis of Association With ROP and Treatment-Warranted ROP in GNN

This supplemental material has been provided by the authors to give readers additional information about their work.

© 2024 Glaser K et al. *JAMA Network Open*.

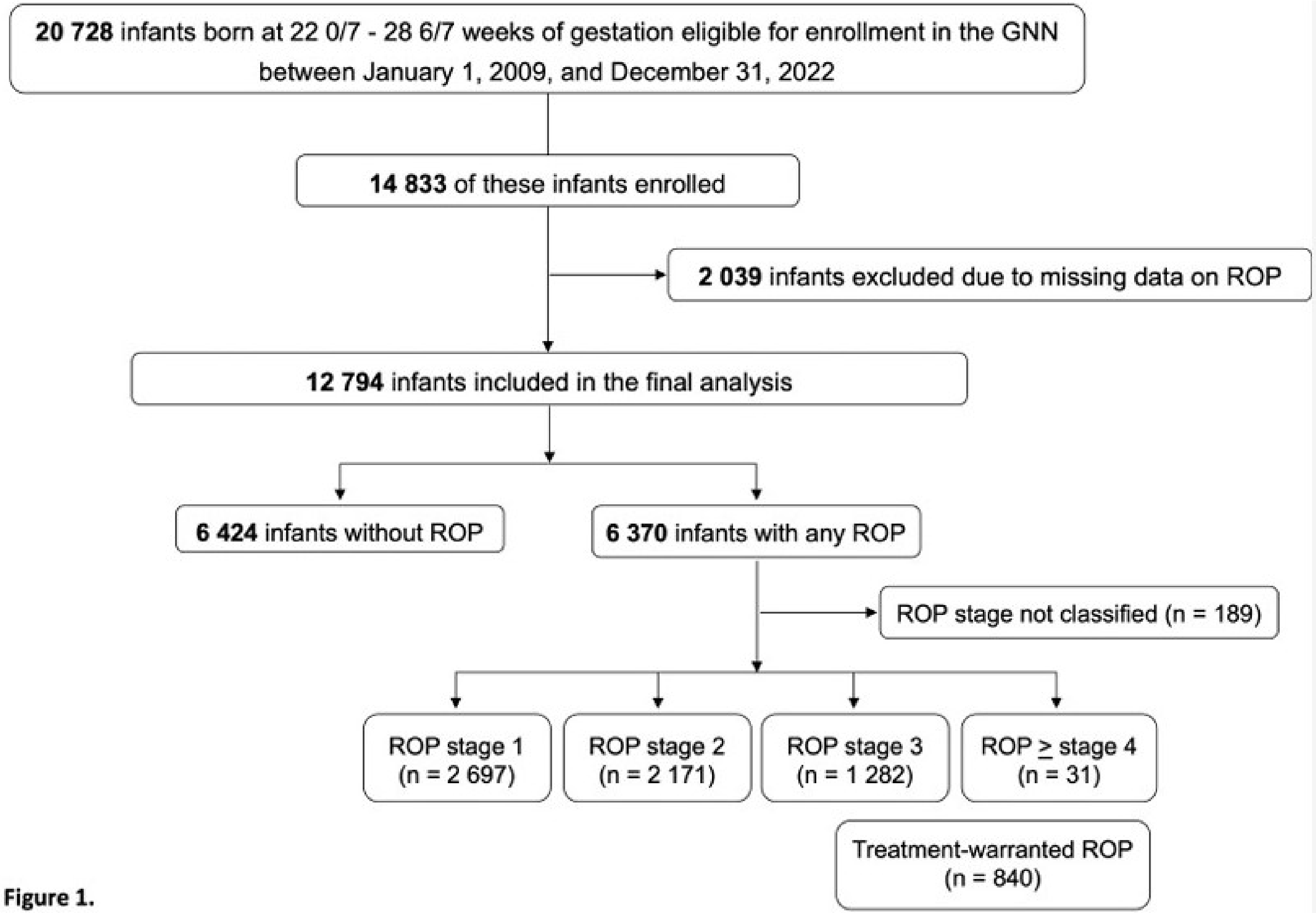

Supplemental Figure 1.
Study Flow Diagram of Infants Enrolled in the German Neonatal Network.

© 2024 Glaser K et al. *JAMA Network Open*.

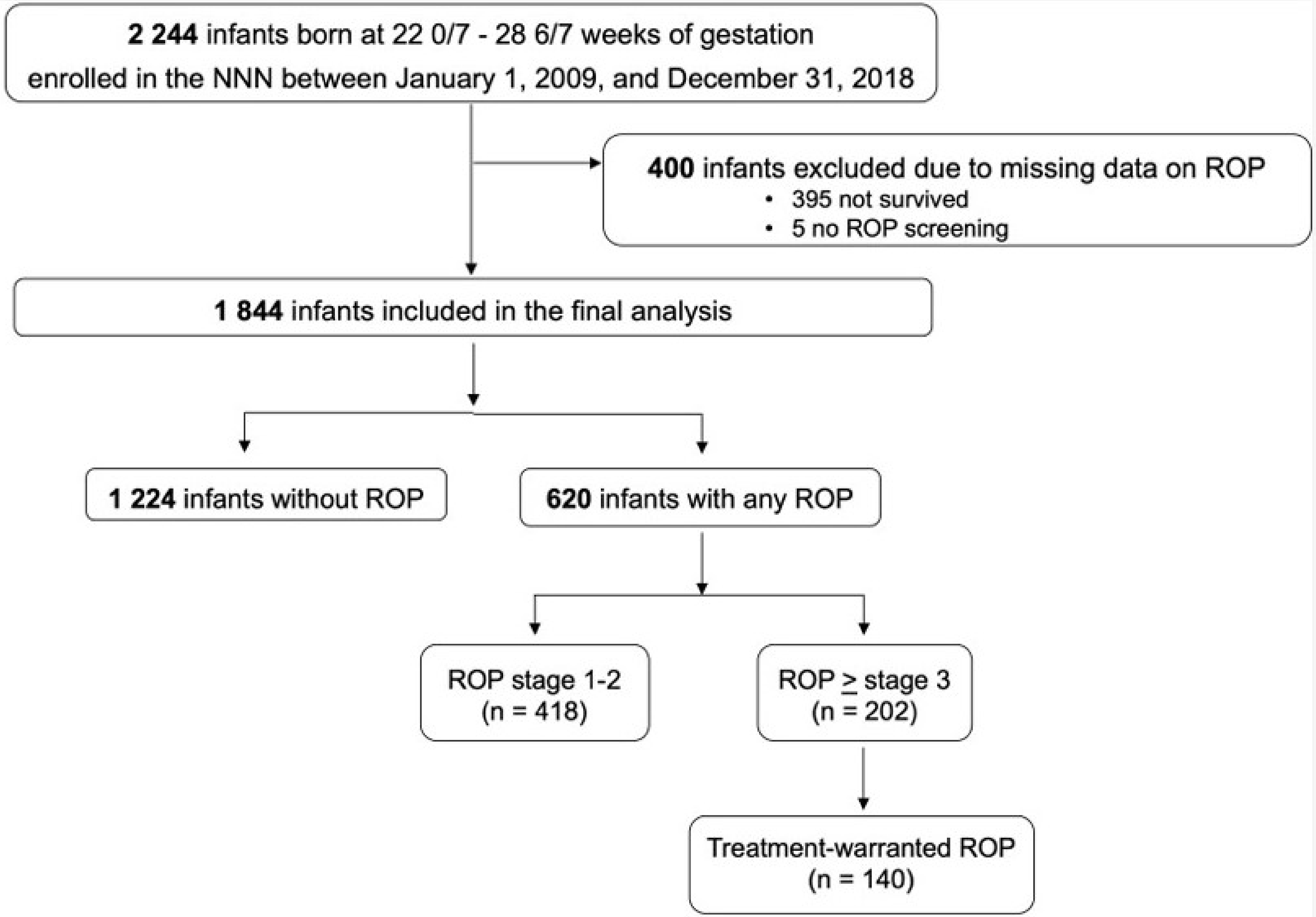

Supplemental Figure 2.
Study Flow Diagram of Infants Enrolled in the Norwegian Neonatal Network.

© 2024 Glaser K et al. *JAMA Network Open*.

eFigure 3. Variables Independently Associated with ROP in GNN in Stepwise Multivariable Regression Analyses

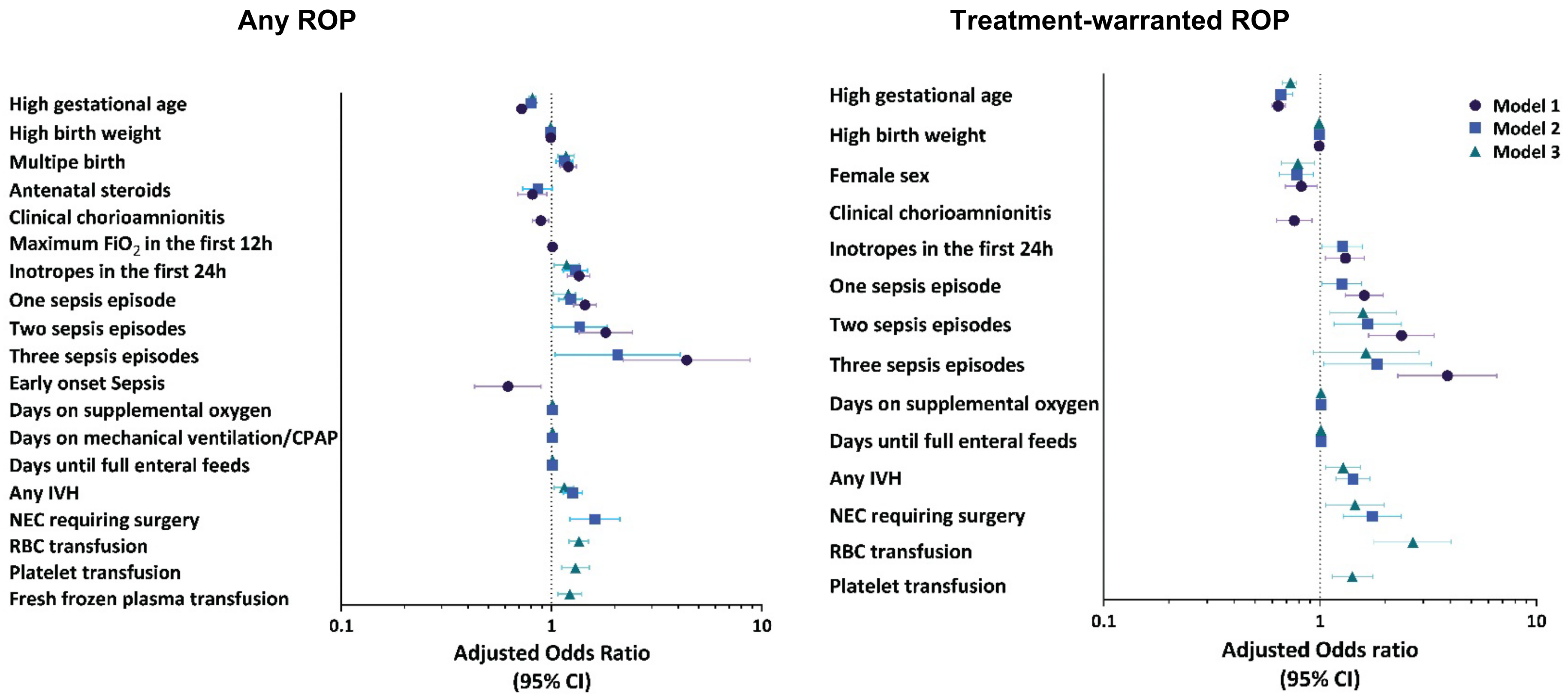

© 2024 Glaser K et al. *JAMA Network Open*.

Multivariable logistic regression modelling testing for an association between neonatal sepsis and any ROP in infants enrolled in the GNN.

Characteristic	Model 1		Model 2		Model 3	
	Adjusted OR (95% CI)	*p* value	Adjusted OR (95% CI)	*p* value	Adjusted OR (95% CI)	*p* value
**High gestational age**	0.72 (0.70 - 0.75)	< .001	0.80 (0.77 - 0.83)	< .001	0.81 (0.78 - 0.84)	< .001
**High birth weight**	0.99 (0.99 - 1.0)	< .001	0.99 (0.99 - 1.0)	< .001	0.99 (0.99 - 1.0)	< .001
**Sex**	0.98 (0.91 - 1.07)	.68	1.09 (0.99 - 1.2)	0.1		
**Multipe birth**	1.20 (1.10 - 1.31)	< .001	1.15 (1.05 - 1.26)	.003	1.17 (1.07 - 1.28)	.001
**Inborn status**	0.93 (0.72 - 1.20)	.57				
**Antenatal steroids**	0.81 (0.69 - 0.95)	.01	0.86 (0.73 - 1.01)	.05	0.87 (0.74 - 1.02)	.09
**Clinical chorioamnionitis**	0.89 (0.81 - 0.97)	.01	0.95 (0.86 - 1.04)	.27		
**Apgar score at 5 min, mean (SD)**	0.99 (0.96 - 1.02)	.47				
**Surfactant replacement via LISA**	1.02 (0.94 - 1.11)	.66				
**Maximum FiO_2 in the first 12h of life**	1.01 (1.0 - 1.01)	< .001	1.01 (1.0 - 1.01)	.09	1.01 (1.01 - 1.02)	.13
**Inotropes given in the first 24h**	1.35 (1.19 - 1.52)	< .001	1.30 (1.14 - 1.48)	< .001	1.18 (1.03 - 1.35)	.02
**Number of sepsis episodes**		< .001		.001		
**One sepsis episode**	1.44 (1.27 - 1.63)	< .001	1.23 (1.08 - 1.40)	.002	1.20 (1.02 - 1.30)	.05
**Two sepsis episodes**	1.81 (1.35 - 2.42)	< .001	1.36 (1.01 - 1.84)	.05	1.27 (0.94 - 1.71)	.12
**Three sepsis episodes**	4.39 (2.19 - 8.78)	< .001	2.06 (1.04 - 4.10)	.04	1.89 (0.96 - 3.75)	.07
**Early onset Sepsis**	0.62 (0.43 - 0.89)	.01	0.78 (0.53 - 1.13)	.19		
**Days on supplemental oxygen**			1.01 (1.0 - 1.01)	< .001	1.01 (1.0 - 1.01)	< .001
**Days on mechanical ventilation or CPAP**			1.01 (1.0 - 1.01)	< .001	1.01 (1.0 - 1.01)	< .001
**Time to achieve full enteral feeding**			1.01 (1.01 - 1.02)	< .001	1.01 (1.0 - 1.01)	< .001
**Any ICH**			1.26 (1.14 - 1.40)	< .001	1.15 (1.03 - 1.27)	.01
**NEC requiring surgery**			1.61 (1.22 - 2.11)	.001	1.28 (0.97 - 1.70)	.08
**RBC transfusion**					1.35 (1.21 - 1.50)	< .001
**Platelet transfusion**					1.30 (1.12 - 1.51)	.001
**Fresh frozen plasma transfusion**					1.22 (1.07 - 1.39)	.002

Abbreviations: CPAP, continuous positive airway pressure; cPVL, cystic periventricular leukomalacia; IVH, intraventricular hemorrhage; NEC, necrotizing enterocolitis; SGA, small for gestational age.

© 2024 Glaser K et al. *JAMA Network Open*.

Multivariable logistic regression modelling testing for an association between neonatal sepsis and treatment-warranted ROP in GNN infants.

Characteristic	Model 1 Adjusted OR (95% CI)	Model 1 *p* value	Model 2 Adjusted OR (95% CI)	Model 2 *p* value	Model 3 Adjusted OR (95% CI)	Model 3 *p* value
High gestational age	0.64 (0.60 - 0.69)	< .001	0.66 (0.64 - 0.75)	< .001	0.73 (0.67 - 0.78)	< .001
High birth weight	0.99 (0.99 - 1.0)	< .001	0.99 (0.99 - 1.0)	< .001	0.99 (0.99 - 1.0)	< .001
Sex	0.82 (0.69 - 0.97)	.02	0.78 (0.65 - 0.93)	.005	0.79 (0.66 - 0.94)	.008
Multipe birth	0.88 (0.73 - 1.05)	.16				
Inborn status	0.99 (0.61 - 1.63)	.99				
Antenatal steroids	1.14 (0.84 - 1.53)	.4				
Clinical chorioamnionitis	0.76 (0.63 - 0.92)	.004	0.86 (0.71 - 1.04)	.12		
Apgar score at 5 min, mean (SD)	0.98 (0.93 - 1.03)	.38				
Surfactant replacement via LISA	0.84 (0.70 - 1.01)	.05	0.88 (0.73 - 1.05)	.16		
Maximum FiO_2 in the first 12h of life	1.01 (0.99 - 1.01)	.15	1.01 (0.99 - 1.01)	.34		
Inotropes given in the first 24h	1.31 (1.06 - 1.60)	.01	1.27 (1.02 - 1.57)	.03	1.15 (0.93 - 1.42)	.20
Number of sepsis episodes		< .001		.004		.03
One sepsis episode	1.60 (1.31 - 1.96)	< .001	1.26 (1.02 - 1.55)	.03	1.14 (0.92 - 1.40)	.24
Two sepsis episodes	2.38 (1.68 - 3.37)	< .001	1.66 (1.16 - 2.38)	.006	1.58 (1.11 - 2.25)	.01
Three sepsis episodes	3.88 (2.29 - 6.55)	< .001	1.84 (1.04 - 3.27)	.04	1.63 (0.93 - 2.87)	.09
Early onset sepsis	0.88 (0.50 - 1.56)	.66				
Days on supplemental oxygen			1.01 (1.0 - 1.01)	< .001	1.01 (1.0 - 1.01)	< .001
Days on mechanical ventilation or CPAP			1.01 (0.99 - 1.01)	.80		
Time to achieve full enteral feeding			1.01 (1.00 - 1.01)	.001	1.01 (0.99 - 1.01)	.10
Any ICH			1.42 (1.19 - 1.70)	< .001	1.28 (1.06 - 1.54)	.009
NEC requiring surgery			1.74 (1.28 - 2.37)	< .001	1.45 (1.06 - 1.98)	.02
RBC transfusion					2.69 (1.78 - 4.05)	< .001
Platelet transfusion					1.41 (1.14 - 1.75)	.001
Fresh frozen plasma transfusion					1.20 (0.98 - 1.47)	.08

Abbreviations: CPAP, continuous positive airway pressure; cPVL, cystic periventricular leukomalacia; IVH, intraventricular hemorrhage; NEC, necrotizing enterocolitis; SGA, small for gestational age.

© 2024 Glaser K et al. *JAMA Network Open*.

eMethods. Description of Models Used in Analysis of Association with ROP and Treatment-Warranted ROP in GNN

Model 1: Multivariable logistic regression analysis included gestational age, birth weight, female sex, multiple birth, inborn status, antenatal steroids, clinical chorioamnionitis, Apgar score at 5min, LISA, maximum FiO_2 in the first 12h of life, inotropes in the first 24h, and sepsis episodes as independent variables.

Model 2: Covariates offered to this model were those variables significant at P < .10 in preceding model 1 (*Any ROP*: gestational age, birth weight, multiple birth, antenatal steroids, clinical chorioamnionitis, maximum FiO_2 in the first 12h, inotropes in the first 24h, early onset sepsis, number of sepsis episodes. *Treatment-warranted ROP*: gestational age, birth weight, female sex, clinical chorioamnionitis, LISA, maximum FIO2 in the first 12h, inotropes given in the first 24h, number of sepsis episodes), plus surrogate markers of poor clinical course comprising days on supplemental oxygen, days on ventilation or CPAP, days until full enteral feeds, any IVH, and NEC requiring surgery.

Model 3: Analyses included all variables significant at P < .10 in preceding model 2 (*Any ROP*: gestational age, birth weight, multiple birth, antenatal steroids, maximum FiO_2 in the first 12h, inotropes in the first 24h, number of sepsis episodes, days on supplemental oxygen, days on ventilation or CPAP, days until full enteral feeds, any IVH, and NEC requiring surgery. *Treatment-warranted ROP*: gestational age, birth weight, female sex, inotropes in the first 24h, number of sepsis episodes, days on supplemental oxygen, any IVH, and NEC requiring surgery), plus, red blood cell transfusions, platelet transfusions, and fresh frozen plasma transfusions.

Alternatively, we excluded the variable birth weight from models 1, 2, and 3. Results obtained from these analyses did not differ from the presented analyses including GA and birth weight.

© 2024 Glaser K et al. *JAMA Network Open*.
