# Supplementary material for: Neonatal Sepsis Episodes and Retinopathy of Prematurity in Very Preterm Infants
Source: JAMA Netw Open. 2024 Jul 25;7(7):e2423933. doi: 10.1001/jamanetworkopen.2024.23933 (PMC11273231; doi:10.1001/jamanetworkopen.2024.23933)
Supplement: Supplement 2. — Nonauthor Collaborators. German Neonatal Network and Norwegian Neonatal Network Investigators [file jamanetwopen-e2423933-s002.pdf]

*Group Name(s): German Neonatal Network and Norwegian Neonatal Network Investigators							
***First Name and Middle Initial(s)**	***Last Name**	***Suffix (eg, Jr, III)**	Academic Degrees	Institution	Location (city, state/province, country)	Role or Contribution, eg, chair, principal investigator	Group (if more than 1 Group listed in the byline) and/or Subgroup (eg, Steering Committee)
Kirstin	Faust		MD	Klinik für Kinder- und Jugendmedizin, UKSH Campus Lübeck	Lübeck	site investigator, patient enrollment	German Neonatal Network
Dirk	Müller		MD	Klinikum Kassel, Kinderklinik - Neonatologie	Kassel	site investigator, patient enrollment	German Neonatal Network
Ulrich	Thome		MD	Universiätsklinikum Leipzig, Klinik für Kinder und Jugendliche	Leipzig	site investigator, patient enrollment	German Neonatal Network
Florian	Guthmann		MD	Kinderkrankenhaus auf der Bult	Hannover	site investigator, patient enrollment	German Neonatal Network
Axel	von der Wense		MD	Altonaer Kinderkrankenhaus e.V.	Hamburg	site investigator, patient enrollment	German Neonatal Network
Christian	Wieg		MD	Klinikum Aschaffenburg-Alzenau, Klinik für Kinder- und Jugendmedizin	Aschaffenburg	site investigator, patient enrollment	German Neonatal Network
Ursula	Weller		MD	Evangelisches Krankenhaus Bielefeld, Klinik für Kinder- und Jugendmedizin	Bielefeld	site investigator, patient enrollment	German Neonatal Network
Thomas	Höhn		MD	Universität Düsseldorf, Klinik für Allgemeine Pädiatrie	Düsseldorf	site investigator, patient enrollment	German Neonatal Network
Dirk	Olbertz		MD	Klinikum Südstadt Rostock, Abteilung für Neonatologie	Rostock	site investigator, patient enrollment	German Neonatal Network
Ursula	Felderhoff-Müser		MD	Universitätskinderklinik Essen	Essen	site investigator, patient enrollment	German Neonatal Network
Rainer	Rossi		MD	Vivantes Krankenhaus Neukölln, Kinder- und Jugendmedizin	Berlin	site investigator, patient enrollment	German Neonatal Network
Norbert	Teig		MD	St. Elisabeth-Hospital, Klinikum der Ruhr-Universiätät Bochum, Klinik für Kinder und Jugendmedizin	Bochum	site investigator, patient enrollment	German Neonatal Network
Friedhelm	Heitmann		MD	Klinikum Dortmund, Klinik für Kinder- und Jugendmedizin	Dortmund	site investigator, patient enrollment	German Neonatal Network

***First Name and Middle Initial(s)**	***Last Name**	***Suffix (eg, Jr, III)**	Academic Degrees	Institution	Location (city, state/province, country)	Role or Contribution, eg, chair, principal investigator	Group (if more than 1 Group listed in the byline) and/or Subgroup (eg, Steering Committee)
Matthias	Heckmann		MD	Ernst-Moritz-Arndt-Universität, Klinik & Poliklinik für Kinder- und Jugendmedizin	Greifswald	site investigator, patient enrollment	German Neonatal Network
Susanne	Schmidtke		MD	Asklepios Klinik Barmbek, Neonatologie	Hamburg	site investigator, patient enrollment	German Neonatal Network
Bettina	Bohnhorst		MD	Medizinische Hochschule Hannover, Kinderklinik I	Hannover	site investigator, patient enrollment	German Neonatal Network
Angela	Kribs		MD	Klinikum der Universität zu Köln, Klinik und Poliklinik für Allgemeine Kinderheilkunde, Neonatologie und Pädiatrische Intensivmedizin	Köln	site investigator, patient enrollment	German Neonatal Network
Matthias	Vochem		MD	Olgahospital Stuttgart, Klinik für Kinder- und Jugendmedizin	Stuttgart	site investigator, patient enrollment	German Neonatal Network
Sven	Wellmann		MD	Kinderklinik St. Hedwig	Regensburg	site investigator, patient enrollment	German Neonatal Network
Jens	Möller		MD	Klinikum Saarbrücken gGmbH, Klinik für Kinder- und Jugendmedizin	Saarbrücken	site investigator, patient enrollment	German Neonatal Network
Joachim	Eichhorn		MD	Klinikum Leverkusen, Klinik für Kinder und Jugendliche	Leverkusen	site investigator, patient enrollment	German Neonatal Network
Jürgen	Wintgens		MD	Städtische Kliniken Mönchengladbach GmbH	Mönchengladbach	site investigator, patient enrollment	German Neonatal Network
Ralf	Böttger		MD	Universitätskinderklinik Magdeburg, Perinatalzentrum	Magdeburg	site investigator, patient enrollment	German Neonatal Network
Markus	Flügel		MD	Ostholstein Kliniken, Kinderklinik Eutin	Eutin	site investigator, patient enrollment	German Neonatal Network
Mechthild	Hubert		MD	DRK-Kinderklinik Siegen, Pädiatrie	Siegen	site investigator, patient enrollment	German Neonatal Network
Michael	Dördelmann		MD	Diakonissenkrankenhaus, Klinik für Kinder- u. Jugendmedizin	Flensburg	site investigator, patient enrollment	German Neonatal Network
Georg	Hillebrand		MD	Klinikum Itzehoe, Kinderklinik	Itzehoe	site investigator, patient enrollment	German Neonatal Network

*First Name and Middle Initial(s)	*Last Name	*Suffix (eg, Jr, III)	Academic Degrees	Institution	Location (city, state/province, country)	Role or Contribution, eg, chair, principal investigator	Group (if more than 1 Group listed in the byline) and/or Subgroup (eg, Steering Committee)
Claudia	Roll		MD	Universität Witten/Herdecke, Vestischen Kinder- und Jugendklinik Datteln	Datteln	site investigator, patient enrollment	German Neonatal Network
Reinhard	Jensen		MD	Westküstenklinikum Heide, Klinik für Kinder- u. Jugendmedizin	Heide	site investigator, patient enrollment	German Neonatal Network
Jens	Möller		MD	Kinderklinik Saarbrücken	Saarbrücken	site investigator, patient enrollment	German Neonatal Network
Mario	Rüdiger		MD	Universitätsklinikum Carl Gustav Carus, Neonatologie u. Intensivmedizin	Dresden	site investigator, patient enrollment	German Neonatal Network
Alexander	Humberg		MD	Kinderklinik der Westfälische Wilhelms-Universität Münster	Münster	site investigator, patient enrollment	German Neonatal Network
Ann Carolin	Longardt		MD	UKSH Campus Kiel, Klinik für Allgemeine Pädiatrie	Kiel	site investigator, patient enrollment	German Neonatal Network
Stefan	Schäfer		MD	Klinikum Nürnberg Süd, Zentrum für Neugeborene, Kinder u. Jugendliche	Nürnberg	site investigator, patient enrollment	German Neonatal Network
Thomas	Schaible		MD	Klinikum Mannheim, Kinderklinik	Mannheim	site investigator, patient enrollment	German Neonatal Network
Axel	Franz		MD	Universitätsklinik für Kinder- und Jugendmedizin Tübingen, Abtlg. Neonatologie	Tübingen	site investigator, patient enrollment	German Neonatal Network
Kay	Hensel		MD	Helios Klinik Wuppertal, Zentrum für Kinder und Jugendmedizin, Neonatologie	Wuppertal	site investigator, patient enrollment	German Neonatal Network
Steffen	Kunzmann		MD	Bürgerhospital, Verein Frankfurter Stiftungskrankenhäuser, Neonatologie	Frankfurt	site investigator, patient enrollment	German Neonatal Network
Esther	Schmidt		MD	HELIOS Klinik Schwerin	Schwerin	site investigator, patient enrollment	German Neonatal Network
Thorsten	Orlikowsky		MD	Universitätsklinikum Aachen, Klinik für Kinder- und Jugendmedizin, Sektion Neonatologie	Aachen	site investigator, patient enrollment	German Neonatal Network

***First Name and Middle Initial(s)**	***Last Name**	***Suffix (eg, Jr, III)**	Academic Degrees	Institution	Location (city, state/province, country)	Role or Contribution, eg, chair, principal investigator	Group (if more than 1 Group listed in the byline) and/or Subgroup (eg, Steering Committee)
Hubert	Gerleve		MD	St. Vinzenzhospital, Kinder- und Jugendklinik	Coesfeld	site investigator, patient enrollment	German Neonatal Network
Nico	Depping		MD	St. Marienhospital Bonn	Bonn	site investigator, patient enrollment	German Neonatal Network
Roland	Haase		MD	Universitätsklinikum Halle, Poliklinik für Kinder- und Jugendmedizin	Halle (Saale)	site investigator, patient enrollment	German Neonatal Network
Marc	Hoppenz		MD	Kliniken der Stadt Köln, Kinderkrankenhaus Amsterdamer Straße - Neonatologie	Köln	site investigator, patient enrollment	German Neonatal Network
Daniel	Vilser		MD	Klinikum St. Elisabeth, Klinik für Kinder- und Jugendmedizin	Neuburg/Donau	site investigator, patient enrollment	German Neonatal Network
Helmut	Küster		MD	Georg-August-Universität Göttingen, Pädiatrische Kardiologie und Intensivmedizin	Göttingen	site investigator, patient enrollment	German Neonatal Network
Hans	Fuchs		MD	Universitätsklinikum Freiburg, Zentrum für Kinder- u. Jugendmedizin Neonatologie / intensivmedizin	Freiburg	site investigator, patient enrollment	German Neonatal Network
Thorsten	Körner		MD	Klinikum Links der Weser GmbH, Klinik für Kinder-und Jugendmedizin, Abt. f. Neonatologie und Pädiatrische Intensivmedizin	Bremen	site investigator, patient enrollment	German Neonatal Network
Thomas	Brune		MD	Klinikum Lippe GmbH, Klinik für Kinder- und Jugendmedizin	Detmold	site investigator, patient enrollment	German Neonatal Network
Andreas	Müller		MD	Universitätsklinikum Bonn (AöR), Zentrum für Kinderheilkunde	Bonn	site investigator, patient enrollment	German Neonatal Network
Florian	Urlichs		MD	St. Franziskus-Hospital Münster, Neonatologie und Kinderintensivmedizin	Münster	site investigator, patient enrollment	German Neonatal Network
Martin	Berghäuser		MD	Florence-Nightingale Krankenhaus, Kinderklinik / Neonatologie und Päd. Intensivmedizin	Düsseldorf	site investigator, patient enrollment	German Neonatal Network

***First Name and Middle Initial(s)**	***Last Name**	***Suffix (eg, Jr, III)**	Academic Degrees	Institution	Location (city, state/province, country)	Role or Contribution, eg, chair, principal investigator	Group (if more than 1 Group listed in the byline) and/or Subgroup (eg, Steering Committee)
Hans	Proquitté		MD	Universitätsklinikum Jena, Klinik für Kinder- und Jugendmedizin, Sektion Neonatologie	Jena	site investigator, patient enrollment	German Neonatal Network
Patrick	Morhart		MD	Universitätsklinikum Erlangen, Neonatologie	Erlangen	site investigator, patient enrollment	German Neonatal Network
Wolfgang	Lindner		MD	Universitätsklinikum Ulm, Klinik für Kinder- und Jugendmedizin	Ulm	site investigator, patient enrollment	German Neonatal Network
Rolf	Schlösser		MD	Universitätsklinik Frankfurt	Frankfurt	site investigator, patient enrollment	German Neonatal Network
Welfhard	Schneider		MD	Vivantes Klinikum am Friedrichshain	Berlin	site investigator, patient enrollment	German Neonatal Network
Michael	Schroth		MD	Cnopf'sche Kinderklinik Nürnberg	Nürnberg	site investigator, patient enrollment	German Neonatal Network
Esther	Rieger-Fackeldey		MD	Klinikum rechts der Isar der Technischen Universität München; Abteilung für Neonatologie und Päd. Intensivmedizin	München	site investigator, patient enrollment	German Neonatal Network
Frank	Dohle		MD	St. Vincenz-Krankenhaus Paderborn Klinik für Kinder- und Jugendmedizin	Paderborn	site investigator, patient enrollment	German Neonatal Network
Jaqueline	Bauer		MD	Klinikum Wolfsburg	Wolfsburg	site investigator, patient enrollment	German Neonatal Network
Florian	Urlichs		MD	Christliches Kinderhospital Osnabrück GmbH Zentrum für Kinder- und Jugendmedizin	Osnabrück	site investigator, patient enrollment	German Neonatal Network
Thomas	Völkl		MD	Klinik für Kinder und Jugendliche Josefinum Augsburg	Augsburg	site investigator, patient enrollment	German Neonatal Network
Francisco	Brevis Nunez		MD	Sana Kliniken Duisburg GmbH	Duisburg	site investigator, patient enrollment	German Neonatal Network
Michael	Welsch		MD	DONAUISAR Klinikum	Deggendorf	site investigator, patient enrollment	German Neonatal Network

*First Name and Middle Initial(s)	*Last Name	*Suffix (eg, Jr, III)	Academic Degrees	Institution	Location (city, state/province, country)	Role or Contribution, eg, chair, principal investigator	Group (if more than 1 Group listed in the byline) and/or Subgroup (eg, Steering Committee)
Marcus	Krüger		MD	München Klinik Harlaching	München Harlaching	site investigator, patient enrollment	German Neonatal Network
				Oslo University Hospital-Rikshospitalet	Oslo		Norwegian Neonatal Network
				Oslo University Hospital-Ullevål	Oslo		Norwegian Neonatal Network
				Akershus University Hospital	Lørenskog		Norwegian Neonatal Network
				Drammen Hospital	Drammen		Norwegian Neonatal Network
				Østfold Hospital Trust	Kalnes		Norwegian Neonatal Network
				Innlandet Hospital Trust	Lillehammer		Norwegian Neonatal Network
				Innlandet Hospital Trust	Elverum		Norwegian Neonatal Network
				Telemark Health Trust	Skien		Norwegian Neonatal Network
				Hospital of Southern Norway	Kristiansand		Norwegian Neonatal Network
				Hospital of Southern Norway	Arendal		Norwegian Neonatal Network
				Stavanger University Hospital	Stavanger		Norwegian Neonatal Network
				Haukeland University Hospital	Bergen		Norwegian Neonatal Network
				Fonna Health Trust	Haugesund		Norwegian Neonatal Network
				Health Sunnmøre Trust	Ålesund		Norwegian Neonatal Network

***First Name and Middle Initial(s)**	***Last Name**	***Suffix (eg, Jr, III)**	Academic Degrees	Institution	Location (city, state/province, country)	Role or Contribution, eg, chair, principal investigator	Group (if more than 1 Group listed in the byline) and/or Subgroup (eg, Steering Committee)
				Førde Health Trust	Førde		Norwegian Neonatal Network
				St. Olav University Hospital	Trondheim		Norwegian Neonatal Network
				Nord-Trøndelag Health Trust	Levanger		Norwegian Neonatal Network
				Nordland Central Hospital	Bodø		Norwegian Neonatal Network
				University Hospital of North Norway	Tromsø		Norwegian Neonatal Network
				Finnmark Health Trust	Hammerfest		Norwegian Neonatal Network
