# Supplementary material for: Neonatal Sepsis Episodes and Retinopathy of Prematurity in Very Preterm Infants
Source: JAMA Netw Open. 2024 Jul 25;7(7):e2423933. doi: 10.1001/jamanetworkopen.2024.23933 (PMC11273231; doi:10.1001/jamanetworkopen.2024.23933)
Supplement: Supplement 3. — Data Sharing Statement [file jamanetwopen-e2423933-s003.pdf]

**Data Sharing Statement**

Glaser. Neonatal Sepsis Episodes and Retinopathy of Prematurity in Very Preterm Infants. *JAMA Netw Open*. Published online July 22, 2024. doi:10.1001/jamanetworkopen.2024.23933

**Data**
**Data available:** Yes
**Data types:** Deidentified participant data
**How to access data:** Data sets are available from W.G. upon reasonable request.
**When available:** With publication

**Supporting Documents**
**Document types:** None

**Additional Information**
**Who can access the data:** To researchers and colleagues requesting the data.
**Types of analyses:** Data sets are available upon reasonable request.
**Mechanisms of data availability:** After approval of a proposal.
